# The clinical and pathological significance of tertiary lymphoid structure in extramammary Paget's disease

**DOI:** 10.3389/fimmu.2024.1435629

**Published:** 2024-11-15

**Authors:** Ningyuan Xi, Xiaoxiang Xu, Mingyuan Xu, Nanhui Wu, Yuhao Wu, Jiashe Chen, Shuyi Liu, Long Jiang, Guorong Yan, Guolong Zhang, Yeqiang Liu

**Affiliations:** 1 Department of Pathology, Shanghai Skin Disease Hospital, School of Medicine, Tongji University, Shanghai, China; 2 Department of Phototherapy, Shanghai Skin Disease Hospital, School of Medicine, Tongji University, Shanghai, China; 3 Department of Dermatologic Surgery, Shanghai Skin Disease Hospital, School of Medicine, Tongji University, Shanghai, China

**Keywords:** extramammary Paget’s disease, tertiary lymphoid structures, histopathology, prognostic significance, skin cancer, non-melanoma skin cancer

## Abstract

**Background:**

Tumor-associated tertiary lymphoid structures (TLSs) are functional immune-responsive aggregates, which have been reported to be associated with better prognosis in various tumors. However, their exact characteristics and prognostic value in extramammary Paget’s disease (EMPD) remain unknown.

**Objective:**

To explore the features of TLSs in EMPD and their association with clinicopathological characteristics.

**Methods:**

In total, 171 EMPD patients from 2015 to 2023, retrospective, single center cohort were collected to assess the presence, maturation status, and location of TLSs by immunohistochemistry. Then, their clinicopathologic association and prognostic significance were further examined.

**Results:**

TLSs were detected in 97 cases (57%) of 171 EMPD patients, including high-density TLSs in 88 cases (91%), peritumoral TLSs (pTLSs) in 89 cases (92%), TLSs around appendages (aTLSs) in 23 cases (24%), and mature TLSs in 16 cases (16%). Secondary EMPD was more likely to produce TLS (Secondary: 16/21 [76%]; Primary: 81/150 [54%]; *P* = 0.06), and more likely to produce Mature TLS (Secondary: 5/10 [50%]; Primary: 11/80 [14%]; *P* = 0.02). The subjective symptoms of EMPD patients did not seem to correlate with the presence of TLS. EMPD patients with tumor invasion were more likely to form mature TLS (Invasion: 8/32 [25%]; *In situ*: 8/65 [12%]; *P* = 0.06), recurrent EMPD patients were more likely to form TLS (Recurrent: 34/50 [68%]; Initial: 63/121 [52%]; *P* = 0.06) especially mature TLS (Recurrent: 8/34 [24%]; Initial: 8/63 [13%]; *P* = 0.04). The depth of tumor invasion in EMPD patients with mature TLS was mostly less than or equal to 4mm (mature TLS+: 7/8 [88%]; TLS-: 6/17 [35%]; *P* = 0.05), aTLS were less common in EMPD patients with skin appendage invasion (aTLS+: 4/23 [17%]; aTLS-: 32/74 [43%]; *P* = 0.03). The same EMPD patients relapse after, the existence of TLS increased [TLS+ (initial): 9/17 (53%); TLS+ (recurrence):14/17 (82%); *P* =.07].

**Limitations:**

Retrospective study design.

**Conclusions:**

Mature TLS is a positive prognostic factor for invasive EMPD and may serve as a new biomarker and therapeutic target for EMPD.

## 1 Introduction

Extramammary Paget’s disease (EMPD) is a rare intraepithelial adenocarcinoma primarily affecting the external genitalia, perianal region, and axillary sweat glands. The typical age of onset ranges from 45 to 75 years. Among Caucasians, EMPD is more common in postmenopausal women, whereas in Asian populations, it predominantly affects men (1). EMPD is characteristically slow-growing and often asymptomatic for prolonged periods, leading to frequent misdiagnosis or delayed diagnosis (2). Most cases have a favorable prognosis, with tumors remaining confined to the epidermis for an extended period as *in situ* lesions (3). However, once subepidermal invasion occurs, there is a significant risk of lymphatic and distant metastasis, which can be life-threatening (4). Surgical excision remains the primary treatment modality for EMPD, but recurrence rates are high, ranging from 30% to 60% (5, 6). Therefore, there is a pressing need for novel therapeutic approaches and reliable prognostic markers to improve treatment outcomes and predict disease prognosis in EMPD.

Tertiary lymphoid structures (TLSs) are ectopic aggregates of immune cells that form in non-lymphoid tissues at sites of chronic inflammation, such as tumors (7), microbial infections (8), graft rejection (9), and autoimmune diseases (10). These structures resemble lymphoid tissue in secondary lymphoid organs (11), containing peripheral populations of T and B lymphocytes, often organized around high endothelial venules (HEVs) (12, 13). TLSs can act as local induction sites for antigen-specific immune responses, allowing the immune system to target antigens within the tissue. The role of TLS in immune regulation is closely linked to its stage of maturation. Immature TLSs are dense aggregates of lymphocytes, primarily composed of randomly distributed B and T cells. Primary TLSs include B cells, T cells, and CD21-positive follicular dendritic cells (FDCs) but lack germinal centers (GCs), while secondary TLSs are more organized, containing CD23-positive GCs (14, 15). TLSs have been associated with clinical outcomes in various cancers, including non-small cell lung cancer, breast cancer, and colorectal cancer (16–18). In skin tumors, such as cutaneous squamous cell carcinoma (cSCC), the presence of TLSs correlates with improved prognosis (11). In melanoma, TLSs are linked to both prognosis and a positive response to PD-1 inhibitors (19). In basal cell carcinoma (BCC), TLSs and the fibrotic matrix play a crucial role in anti-tumor immunity (20). However, the presence, characteristics, and clinical significance of TLSs in EMPD remain unclear.

In the treatment of EMPD, traditional approaches often focus on targeting tumor cells directly but may not achieve satisfactory outcomes. Recently, tumor immunotherapy has shown significant therapeutic potential by stimulating the patient’s immune system to combat tumors (11, 21). The tumor microenvironment (TME) plays a critical role in the progression of malignancies, encompassing both the host’s anti-tumor immune response and the destruction of normal tissues. Tumor-infiltrating lymphocytes (TILs), a key component of the TME, are indicative of the host’s immune response to the tumor, with their abundance often associated with favorable prognosis. The interaction between tumor cells and immune cells is essential for tumor initiation, local invasion, and metastasis (22, 23). A high density of TILs is generally considered a positive prognostic factor. TLS act as functional immune niches, facilitating the recruitment and activation of T cells, mediating an effective anti-tumor immune response, and promoting a favorable lymphoid neovascularization environment within tumor tissues. This, in turn, enhances local T cell responses and contributes to tumor regression. Consequently, inducing the formation of TLSs within the TME represents a promising strategy for improving anti-tumor immunity in EMPD.

Given the critical role of TLS in anti-tumor immunity, it is essential to assess TLS in the context of EMPD. This study aims to investigate the presence and characteristics of TLS in EMPD and explore the relationship between TLS and the clinicopathological features of the disease.

## 2 Methods

### 2.1 Estimation of the IME composition of EMPD

Transcriptomic data with a total of eight samples including four EMPD and four normal skin (NS) tissues were downloaded from the Gene Expression Omnibus (GSE117285). The immune microenvironment (IME) of each sample was assessed by the MCP-counter R-package, with default parameters. Furthermore, the expressions of chemokines were also investigated using limma R-package. *P*-value was adjusted by false discovery rate. Gene with | log2 (fold change) | > 1 and an adjusted P < 0.05 was considered as a differential expression (24).

### 2.2 Data collection

A total of 171 EMPD patients who underwent histological validation by two independently certified pathologists at Shanghai Skin Disease Hospital from 2015 to 2023 were retrospectively selected for this study. All patients have H&E staining slides, formalin fixed paraffin embedded blocks and complete clinical information. No patients received any chemotherapy or radiation before surgery. The human samples used in this study have been approved by the ethics committee of Shanghai Skin Disease Hospital. The title of the ethical project is clinicopathological analysis of tertiary lymphoid structure in EMPD and its use as a prognostic marker. The reference number of the approval is 2023-06. We reviewed clinical and histopathological data from patient files. All relevant data is obtained anonymously.

### 2.3 Immunohistochemistry (IHC)

Immunohistochemical staining was performed on the markers of CD3 (T cells, ab16669, Abcam, 1:150), CD20 (B cells, ab64088, Abcam, 1:100), CD21 (Follicular DC, ab7290, Abcam, 1:20) to investigate the presence of TLS. Specifically, the samples were deparaffinized in xylene and rehydrated in a graded alcohol bath. The samples were then incubated in 3% H2O2 for 15 min. Heat-induced antigen repair was performed in sodium citrate buffer at pH 6.0. Place the slide in 5% Bovine Serum Albumin at 37°C for 30 min. The experimental group was instilled with primary antibody, and the control group was instilled with phosphate buffer solution. The samples were incubated at 4°C overnight. After that, the samples were incubated with secondary antibodies at 37°C for 15 min, stained with diamine benzidine for 2 to 6 min depending on the type of antibody, and then stained with hematoxylin. The cells were differentiated in 1% hydrochloric ethanol, dehydrated in xylene and graded alcohol baths, sealed, and observed.

For our negative control, Phosphate Buffered Saline was used instead of the primary antibody during IHC staining, and the other procedures were the same as for the experimental group. Lymph node tissues containing CD3-positive T cells, CD20 B cells and CD21-positive FDC cells were also used as positive control.

### 2.4 Pathological examination and immunohistochemical evaluation

Pathological examination was conducted by two independent certified pathologists who were unaware of the patient’s clinical characteristics to identify the H&E stained sections and immunohistochemical sections of EMPD patients. H&E staining sections was used to observe the number and maturity of lymphocyte aggregates, the presence of skin appendages, and the presence of tumor invasion *in situ*. According to the maximum number of TLSs visible under the 40X magnification field of the microscope, it can be divided into three groups: TLS negative group (without TLS), TLS low-density group (one TLS), and TLS high-density group (multiple TLSs). The diagnosis of EMPD is firstly based on clinical presentation, such as infiltrative erythematous plaques, and primarily confirmed through histopathological findings, particularly the presence of intraepidermal Paget cells. Immunohistochemical markers such as CEA, CK7, and EMA are used to differentiate EMPD from other conditions and establish a diagnosis of primary EMPD. In cases where CK20 is positive and CK7 is negative, a diagnosis of secondary EMPD is made.

TLS were classified as mature or immature based on CD21-positive FDCs. According to its relative position to the tumor, if TLS is less than or equal to 500 μm from the tumor and is not surrounded by tumor cells, it is called peritumoral TLS (pTLS). If TLS is surrounded by tumor cells, it is intratumoral TLS (iTLS), and if the distance from the tumor is greater than 500μm, it is stromal TLS (sTLS). If multiple TLS were present and included pTLS, they were considered pTLS. According to the relative position of TLS and skin appendages, TLS were divided into TLS around the skin appendages(aTLS) and TLS far away from the skin appendages(fTLS). If more than one TLS exists and contains aTLS, they are considered aTLS.

At present, there is no authoritative tumor staging plan for EMPD, but there is a literature that has established the staging of EMPD (25). To better explore the influence of TLS on the prognosis of EMPD, we performed the staging of EMPD on this basis. Stage I is an *in-situ* tumor only, stage II is an aggressive tumor with a tumor thickness ≤ 4mm and no lymphatic or vascular invasion, stage III is an aggressive tumor with a tumor thickness > 4mm and no lymphatic or vascular invasion, and stage III has lymphatic or vascular invasion regardless of *in-situ*, invasion and tumor thickness. Because there is no authoritative tumor staging scheme for EMPD at present, and there is no statistical difference in our study results, we did not write the results in the paper.

### 2.5 Statistical analysis

Statistical analysis was conducted using IBM SPSS STATISTICS 22.0. Chi-square test, continuity correction chi-square test or Fisher test were used to compare the relationship between TLS expression and clinicopathological parameters. *P* value < 0.05 is considered statistically significant.

## 3 Results

### 3.1 TLS characteristics in EMPD

**Figure 1:**
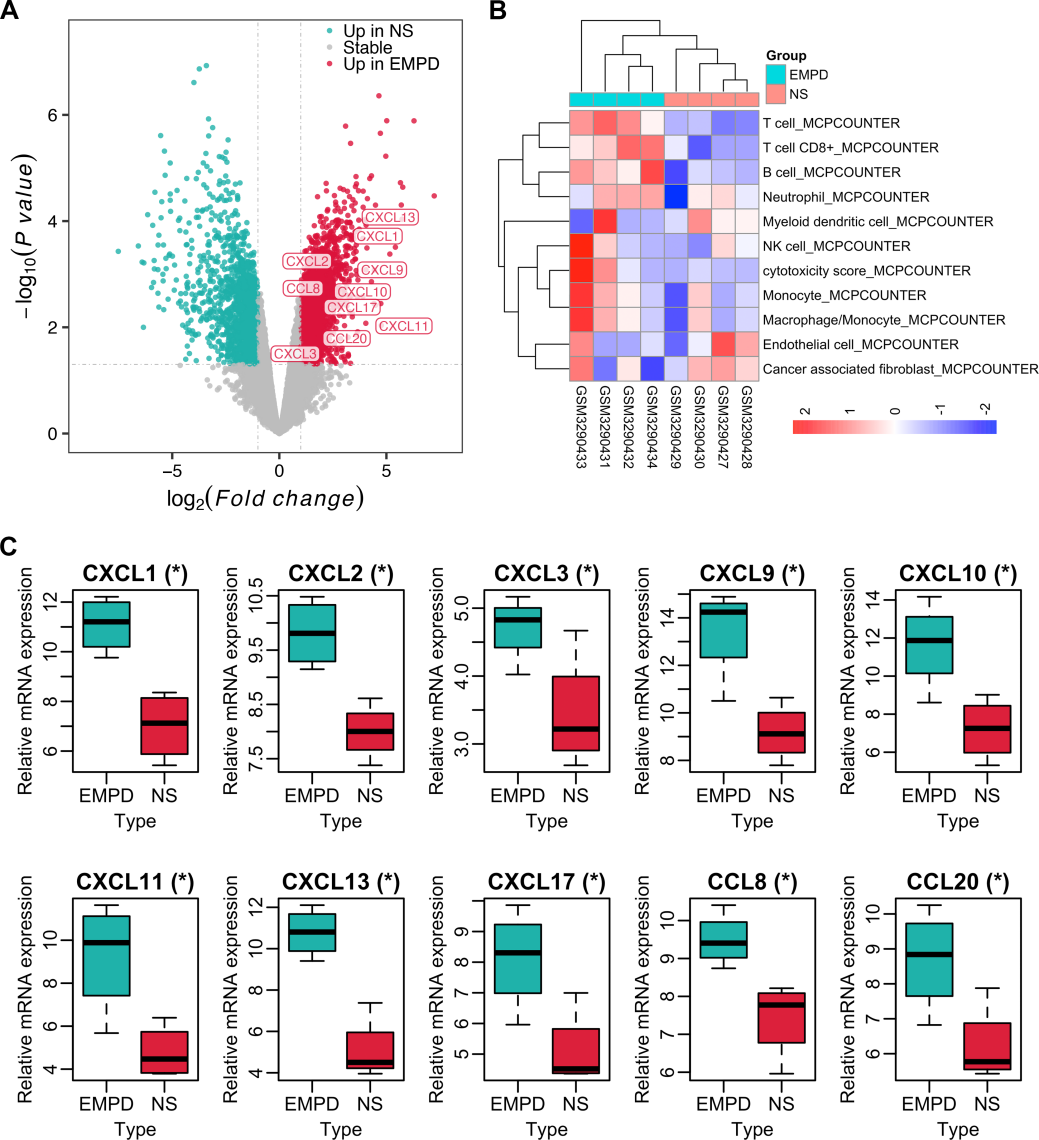
IME composition of EMPD and NS tissues in GSE117285. **(A)** Volcano plot for differentially expressed analysis in EMPD *vs*. NS tissue. **(B)** The IME composition in EMPD and NS tissues analyzed by the MCP-counter. NK cells, natural killer cells. **(C)** Differential expression of TLS-related chemokines in EMPD and NS tissue. NS, normal skin.

The IME composition estimation analysis revealed that the IME composition was significantly different between EMPD and NS tissues (**Figure 1A**). Specifically, most EMPD samples were significantly infiltrated with B cells, monocyte lineages, T cells, cytotoxic lymphocytes, CD8 T cells, and natural killer (NK) cells (**Figure 1B**). Furthermore, various chemokines, such as CXCL13, were significantly up regulated in EMPD tissues (**Figure 1C**). Collectively, the bioinformatics results showed the potential presence of TLS in EMPD. Then, we further investigate the detailed characteristics of TLS in EMPD.

Among the 171 patients in our study, 21 were younger than 60 years (21/171), and 146 were male (146/171). The most common site of initial onset was the scrotum, affecting 148 patients (148/171). Clinically, erythema (164/171) and erosion (72/171) were the most frequently observed symptoms, whereas ulcers (14/171), hypopigmentation (41/171) and nodules (15/171) were less common. Most patients had primary EMPD (150/171), and most presented with *in situ* lesions (122/171). Itching was the most common symptom (119/171), followed by pain (48/171). Appendicular involvement was seen in 56 patients (56/171), and the majority were newly diagnosed cases (121/171). A smaller subset of patients exhibited vascular or lymphatic infiltration (27/171). Among the 49 patients for whom invasion depth was assessed, nearly half had a depth of invasion greater than 4 mm (25/49).

**Figure 2:**
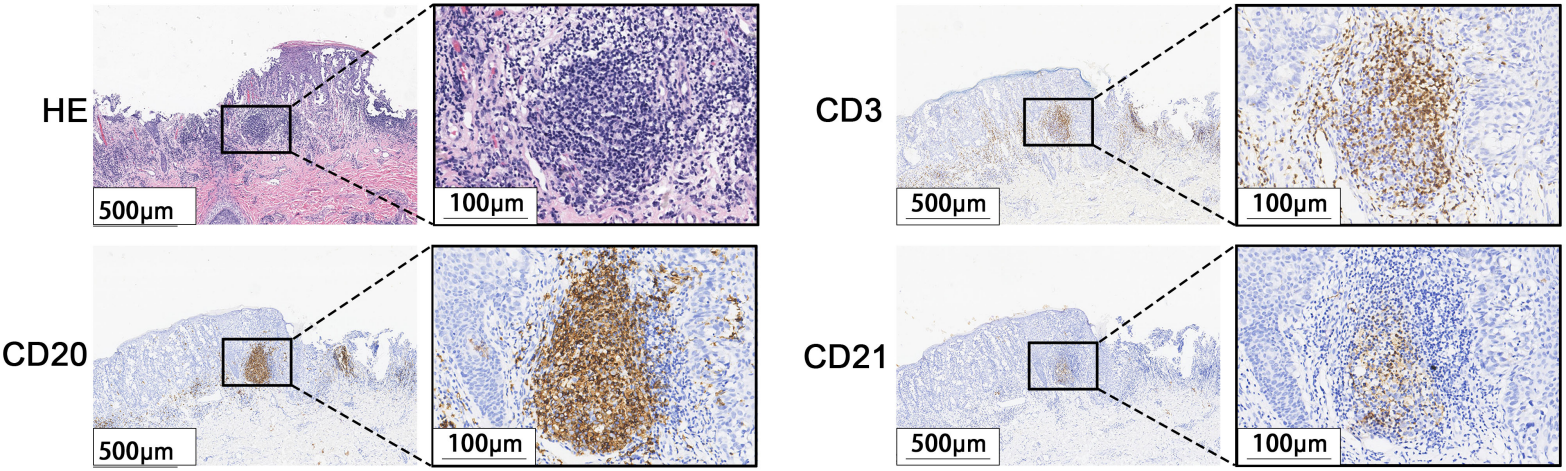
Characteristics of TLS. H&E stained sections staining showed lymphocyte aggregation. Immunohistochemical staining showed that the center was CD20-positive B lymphocytes, the surrounding CD3-positive T lymphocytes, and the GC was CD21-positive FDCs.

**Figure 3:**
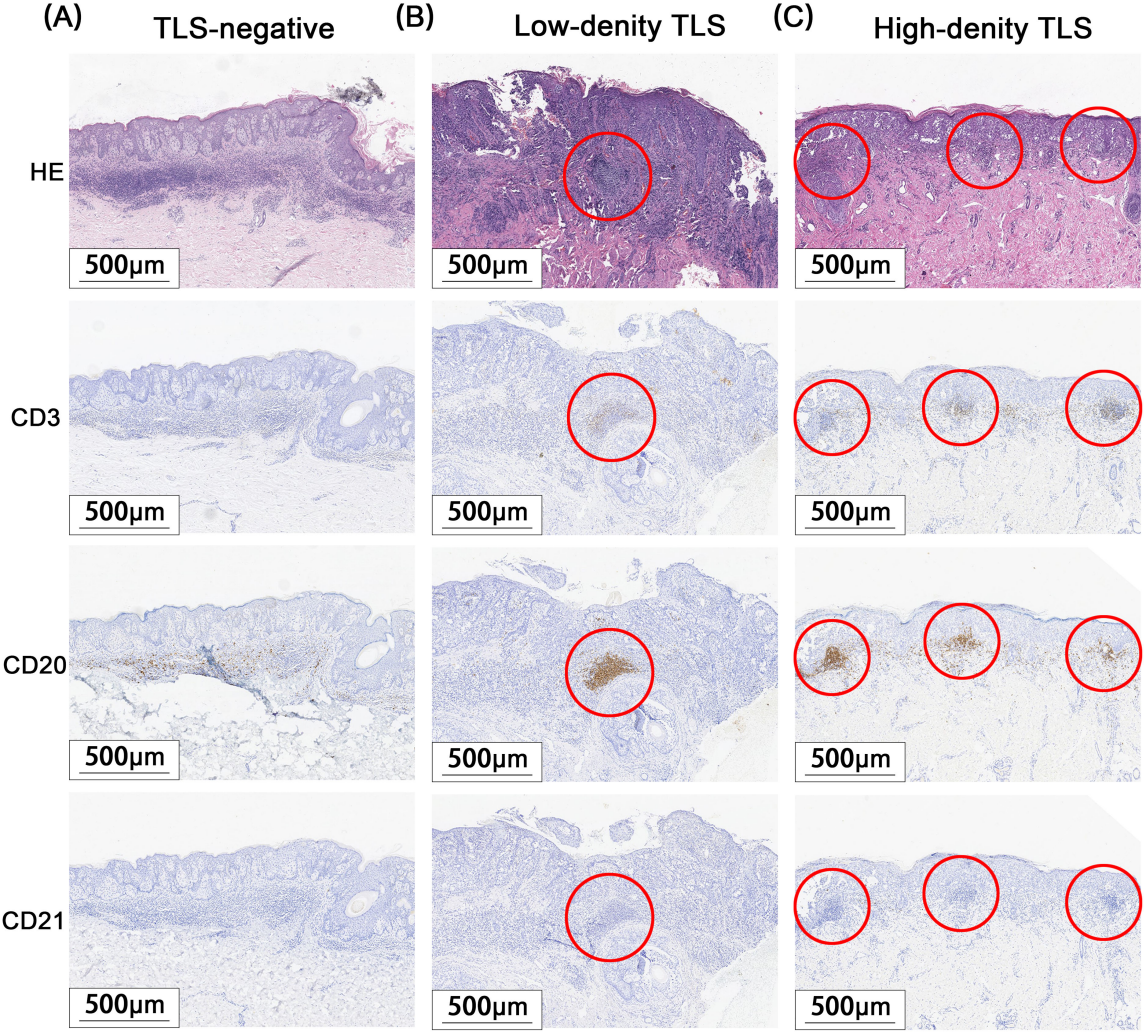
Different Density TLS. Red circle areas show representative TLSs. **(A)** TLS negative group. **(B)** TLS low-density group. **(C)** TLS high-density group.

**Figure 4:**
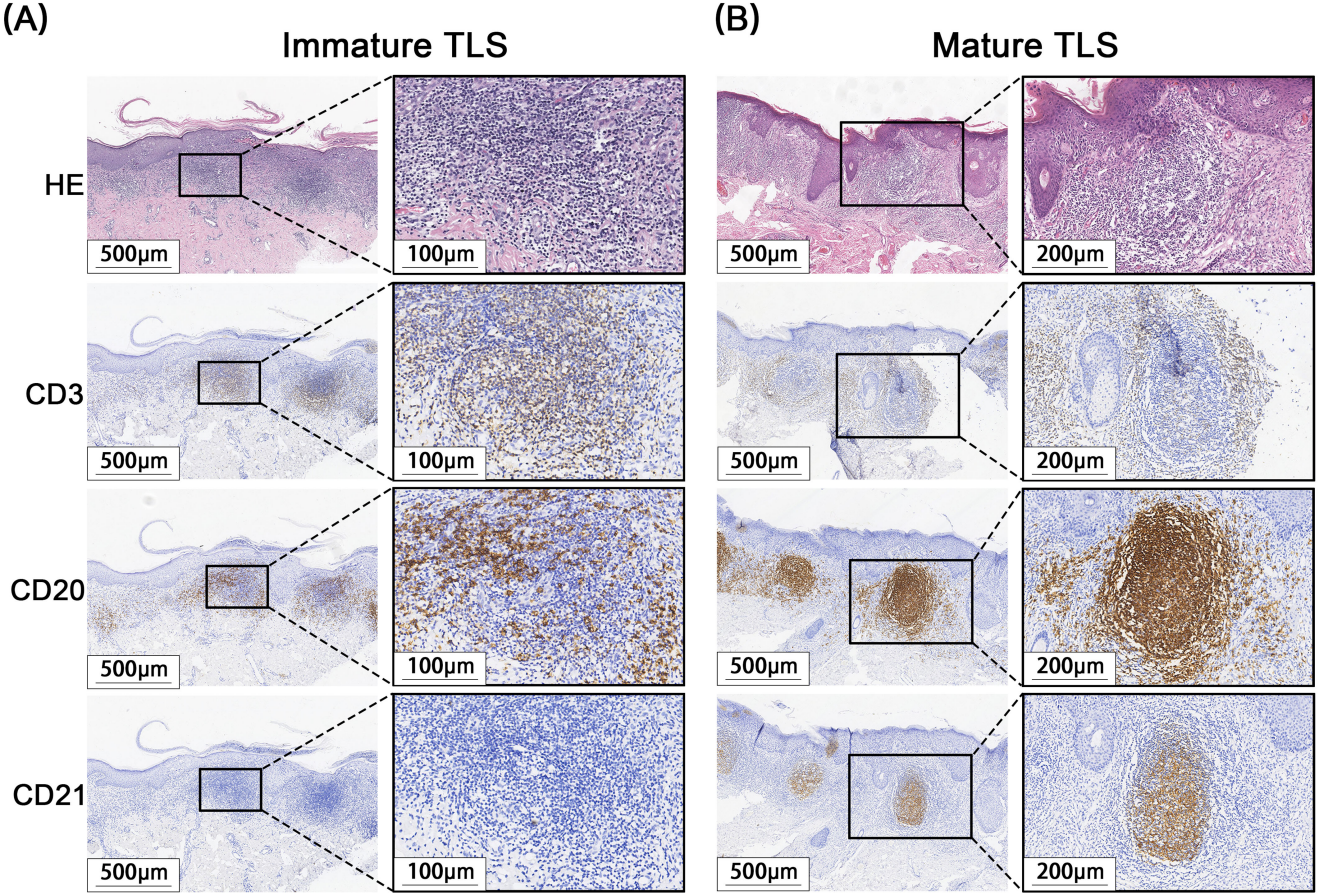
Different maturations of TLS. **(A)** Immature TLS: The germinal center CD21 is negative, and no obvious follicle-like structure is observed. **(B)** Mature TLS: CD21 positive in the germinal center, with obvious follicle-like structures visible.

**Figure 5:**
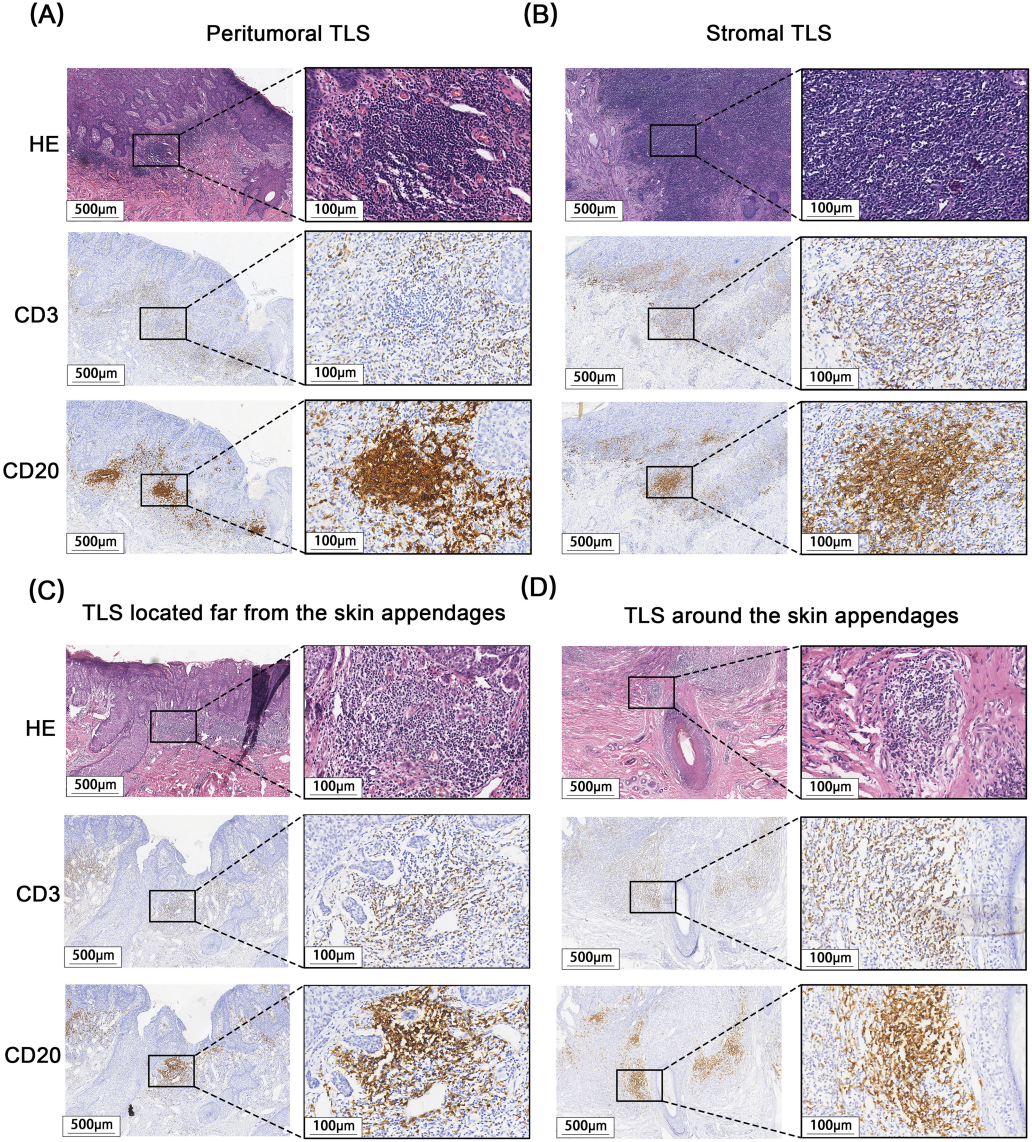
Different Relative location of TLS to tumor and appendages. **(A)** pTLS, Peritumoral TLS. **(B)** sTLS, Stromal TLS. **(C)** fTLS, TLS located far from the skin appendages. **(D)** aTLS, TLS around the skin appendages.

A total of 171 cases of EMPD met the inclusion criteria for this study. H&E staining sections showed that TLS often exhibited lymphocyte aggregation. Within these structures, CD20+ B lymphocytes were located at the center, while CD3+ T lymphocytes were found at the periphery. Mature TLS also contained structures formed by CD21+ follicular dendritic cells (FDCs) (**Figure 2**). IHC analysis identified TLS in 97 cases of EMPD (57%). Among these, 88 cases (91%) exhibited high-density TLS, while 9 cases (9%) showed low-density TLS (**Figure 3**). Regarding TLS maturation, only 16 cases of EMPD exhibited CD21+ TLS (**Figure 4**). Furthermore, 89 cases (91.8%) were classified as pTLS, whereas 23 cases (23.7%) were classified as aTLS (**Figure 5**).

### 3.2 The relationship between TLS and clinicopathological features

The baseline clinicopathological features and outcomes of the cohort are summarized in **Tables 1**, **2**. Secondary EMPD was more likely to produce tertiary lymphoid structures (TLS), with 16 of 21 secondary cases (76%) showing TLS compared to 81 of 150 primary cases (54%; *P* = 0.06). Additionally, secondary EMPD cases were more likely to have mature TLS (5 of 16 secondary cases [31%] vs. 11 of 81 primary cases [14%]; *P* = 0.02). Subjective symptoms did not correlate with the presence of TLS. Patients with tumor invasion were more likely to develop mature TLS, with 8 of 32 invasive cases (25%) exhibiting mature TLS compared to 8 of 65 *in situ* cases (12%; *P* = 0.06). Recurrent EMPD patients were also more likely to form TLS (34 of 50 recurrent cases [68%] vs. 63 of 121 initial cases [52%]; *P* = 0.06), especially mature TLS (8 of 34 recurrent cases [24%] vs. 8 of 63 initial cases [13%]; *P* = 0.04). Most EMPD patients with mature TLS had tumor invasion depths of 4 mm or less (7 of 8 cases [88%] with mature TLS vs. 6 of 17 cases [35%] without mature TLS; *P* = 0.05). aTLS were more less in patients with skin appendage invasion (4 of 23 cases [17%] with aTLS vs. 32 of 74 cases [43%] without aTLS; *P* = 0.03). The pathological characteristics of the baseline cohort of EMPD patients before and after recurrence are showed in **Table 3**. Finally, among the same EMPD patients who relapsed, the presence of TLS increased from 9 of 17 initial cases (53%) to 14 of 17 recurrent cases (82%; *P* = 0.07).

**Table 1:** Correlation analysis between TLS presence and maturity and clinicopathological features of EMPD patients.

	Presence			TLS maturity			TLS maturity		
	TLS (n = 97)	No TLS (n = 74)	P*/OR	Immature TLS (n = 81)	No TLS (n = 74)	P*/OR	Mature TLS (n = 16)	No TLS (n = 74)	P*/OR
Age, n (%)			.37/1.52			.34/1.59			1$/1.22
≥60	87 (90)	63 (85)		73 (90)	63 (85)		14 (86)	63 (85)	
<60	10 (10)	11 (15)		8 (10)	11 (15)		2 (14)	11 (15)	
Sex, n (%)			.94/1.04			.82/1.11			.99$/0.76
Male	83 (86)	63 (85)		70 (86)	63 (85)		13 (81)	63 (85)	
Female	14 (14)	11 (15)		11 (14)	11 (15)		3 (19)	11 (15)	
Location, n (%)^a^									
Scrotum	86 (89)	62 (84)	.35/1.51	71 (88)	62 (84)	.49/1.37	15 (94)	62 (84)	.53$/2.90
Armpit	1 (1)	6 (8)	.054$/0.12	1 (1)	6 (8)	.10$/0.14	0 (0)	6 (8)	.53$/N/A
Perianal	7 (7)	5 (7)	.91/1.07	6 (7)	5 (7)	.88/1.10	1 (6)	5 (7)	1$/0.92
Other sites	3 (3)	1 (1)	.81$/2.33	3 (4)	1 (1)	.68$/2.81	0 (0)	1 (1)	1#/N/A
Clinical findings, n (%)									
Erythema	92 (95)	72 (97)	.68$/0.51	78 (96)	72 (97)	1$/0.72	14 (86)	72 (97)	.14#/0.19
No erythema	5 (5)	2 (3)		3 (4)	2 (3)		2 (14)	2 (3)	
Erosions	36 (37)	36 (49)	.13/0.62	30 (37)	36 (49)	.14/0.62	6 (38)	36 (49)	.42/0.63
No erosions	61 (63)	38 (51)		51 (63)	38 (51)		10 (62)	38 (51)	
Ulcers	7 (7)	7 (9)	.60/0.74	5 (6)	7 (9)	.44/0.63	2 (14)	7 (9)	1$/1.37
No ulcers	90 (93)	67 (81)		76 (94)	67 (81)		14 (86)	67 (81)	
Hypopigmentation	26 (27)	15 (20)	.32/1.44	23 (28)	15 (20)	.24/1.56	3 (19)	15 (20)	1$/0.91
No hypopigmentation	71 (73)	59 (80)		58 (72)	59 (80)		13 (81)	59 (80)	
Nodules	8 (8)	7 (9)	.78/0.86	5 (6)	7 (9)	.44/0.63	3 (19)	7 (9)	.53$/2.21
No nodules	89 (92)	67 (91)		76 (94)	67 (91)		13 (81)	67 (91)	
Primary/Secondary, n (%)			.06/0.37			.16/0.46			.02*$/0.16
Primary	81 (84)	69 (93)		70 (86)	69 (93)		11 (69)	69 (93)	
Secondary	16 (16)	5 (7)		11 (14)	5 (7)		5 (31)	5 (7)	
Subjective symptom, n (%)^b^									
Only Itching	55 (57)	51 (69)	.33/0.59	47 (58)	51 (69)	.28/0.55	8 (50)	51 (69)	1#/0.94
Only pain	22 (23)	13 (18)	.90/0.92	17 (21)	13 (18)	.70/0.78	5 (31)	13 (18)	.85$/2.31
Itching and pain	9 (9)	4 (5)	1$/1.23	7 (9)	4 (5)	1$/1.05	2 (13)	4 (5)	.88$/3.00
No subjective symptoms	11 (11)	6 (8)		10 (12)	6 (8)		1 (6)	6 (8)	
Tumor *In situ* or invasive tumor, n (%)			.15/0.61			.35/0.71			.06$/0.30
in situ	65 (67)	57 (77)		57 (70)	57 (77)		8 (50)	57 (77)	
Invasion	32 (33)	17 (23)		24 (30)	17 (23)		8 (50)	17 (23)	
Appendicular involvement, n (%)			.16/1.59			.24/1.51			.31$/2.10
Yes	36 (37)	20 (27)		29 (36)	20 (27)		7 (44)	20 (27)	
No	61 (63)	54 (73)		52 (64)	54 (73)		9 (56)	54 (73)	
Initial/recurrence, n (%)			.06/0.51			.14/0.58			.04*$/0.28
Initial	63 (65)	58 (78)		55 (68)	58 (78)		8 (50)	58 (78)	
Recurrence	34 (35)	16 (22)		26 (32)	16 (22)		8 (50)	16 (22)	
Vascular/lymphatic infiltration, n (%)			.48/1.36			.52/1.34			.88$/1.48
Yes	17 (18)	10 (14)		14 (17)	10 (14)		3 (19)	10 (14)	
No	80 (82)	64 (86)		67 (83)	64 (86)		13 (81)	64 (86)	
Depth of invasion, n (%)	n = 32	n = 17	.16/2.36	n = 24	n = 17	.50/1.55	n = 8	n = 17	.05*$/12.83
≤4mm	18 (56)	6 (35)		11 (46)	6 (35)		7 (88)	6 (35)	
>4mm	14 (44)	11 (65)		13 (54)	11 (65)		1 (12)	11 (65)	

*P* values based on chi square unless otherwise specified.

^$^*P*-value based on Continuity correction chi square test.

^#^*P*-value based on Fisher’s exact.

OR, Odds Ratio.

a The control group was all the onset sites except the experimental group.

b The control group was the non-subjective symptom group.

*P ≤ 0.05.

**Table 2:** Correlation analysis between TLS location and clinicopathological features of EMPD patients.

	TLS location to the tumor			TLS location to the appendage		
	Peritumoral (n = 89)	Stromal (n = 8)	P*/OR	Around appendage (n = 23)	far from appendage (n = 74)	P*/OR
Age, n (%)			1#/1.27			.38$/0.42
≥60	80 (90)	7 (88)		19 (83)	68 (92)	
<60	9 (10)	1 (12)		4 (17)	6 (8)	
Sex, n (%)			.72$/2.14			1$/1.16
Male	77 (87)	6 (75)		20 (87)	63 (85)	
Female	12 (13)	2 (25)		3 (13)	11 (15)	
Location, n (%)^a^						
Scrotum	79 (89)	7 (87)	1#/1.13	21 (26)	65 (60)	.94$/1.45
Armpit	1 (1)	0 (0)	1#/N/A	0 (0)	1 (1)	1#/N/A
Perianal	6 (7)	1 (13)	.46#/0.51	1 (4)	6 (8)	.88$/0.52
Other sites	3 (3)	0 (0)	1#/N/A	1 (4)	2 (3)	.56#/1.64
Clinical findings, n (%)						
Erythema	85 (96)	7 (87)	.36#/3.04	22 (96)	70 (95)	1$/1.26
No erythema	4 (4)	1 (13)		1 (4)	4 (5)	
Erosions	33 (37)	3 (38)	1$/0.98	9 (39)	27 (36)	.82/1.12
No erosions	56 (63)	5 (62)		14 (61)	47 (64)	
Ulcers	6 (7)	1 (13)	.46#/0.51	2 (9)	5 (7)	1$/1.31
No ulcers	83 (93)	7 (87)		21 (91)	69 (93)	
Hypopigmentation	25 (28)	1 (13)	.59$/2.73	6 (26)	20 (27)	.93/0.95
No hypopigmentation	64 (72)	7 (87)		17 (74)	54 (73)	
Nodules	7 (8)	1 (13)	.51#/0.60	2 (9)	6 (8)	1$/1.08
No nodules	82 (92)	7 (87)		21 (91)	68 (92)	
Primary/Secondary, n (%)			.86$/1.79			.85$/1.42
Primary	75 (84)	6 (75)		20 (87)	61 (82)	
Secondary	14 (16)	2 (25)		3 (13)	13 (18)	
Subjective symptom, n (%)^b^						
Only Itching	51 (57)	4 (50)	1#/1.28	12 (52)	43 (58)	.21$/0.33
Only pain	20 (23)	2 (25)	1$/1.00	3 (13)	19 (26)	.11$/0.19
Itching and pain	8 (9)	1 (12.5)	1#/0.80	3 (13)	6 (8)	.93$/0.60
No subjective symptoms	10 (11)	1 (12.5)		5 (22)	6 (8)	
Tumor *In situ* or invasive tumor, n (%)			.91$/0.66			.47/0.70
in situ	59 (66)	6 (75)		14 (61)	51 (69)	
Invasion	30 (34)	2 (25)		9 (39)	23 (31)	
Appendicular involvement, n (%)			1$/0.98			.03*/0.28
Yes	33 (37)	3 (37)		4 (17)	32 (43)	
No	56 (63)	5 (63)		19 (83)	42 (57)	
Initial/recurrence, n (%)			.31$/0.24			.13/2.32
Initial	56 (63)	7 (88)		18 (78)	45 (61)	
Recurrence	33 (37)	1 (12)		5 (22)	29 (39)	
Vascular/lymphatic infiltration, n (%)			1$/1.53			.36$/2.02
Yes	16 (18)	1 (13)		6 (26)	11 (15)	
No	73 (82)	7 (87)		17 (74)	63 (85)	
Depth of invasion, n (%)	n = 30	n = 2	1#/1.31	n = 9	n = 23	1$/0.96
≤4mm	17 (57)	1 (50)		5 (56)	13 (57)	
>4mm	13 (43)	1 (50)		4 (44)	10 (43)	

*P* values based on chi square unless otherwise specified.

^$^*P*-value based on Continuity correction chi square test.

^#^*P*-value based on Fisher’s exact.

OR, Odds Ratio.

a The control group was all the onset sites except the experimental group.

b The control group was the non-subjective symptom group.

*P ≤ 0.05.

**Table 3:** Correlation between primary and recurrent disease and TLS characteristics in EMPD patients.

	Site		
	Initial	Recurrent	P^*^/OR
TLS density, n (%)			
Negative	8 (53)	3 (18)	
Low	0	0	.07/4.15
High	9 (47)	14 (82)	
TLS maturity^$^, n (%)			
Mature	1 (11)	2 (22)	1$/0.44
Immature	8 (89)	7 (78)	
Relative location of TLS to tumor^#^, n (%)			
pTLS	9 (100)	8 (89)	1#/N/A
sTLS	0 (0)	1 (11)	
Relative location of TLS to appendages^$^, n (%)			
Around the appendages	3 (33)	5 (56)	.64$/0.40
Located far from the appendage	6 (67)	4 (44)	

*P* values based on chi square unless otherwise specified.

^$^*P*-value based on Continuity correction chi square test.

^#^*P*-value based on Fisher’s exact.

OR, Odds Ratio.

*P ≤ 0.05.

## 4 Discussion

To the best of our knowledge, this is the first study to evaluate the spatial location, density, and maturity of TLS in EMPD. Our results show a moderate TLS positivity rate in EMPD, at 57%. TLS has been observed in various tumors, with reported rates of 47% in hepatocellular carcinoma (26), 43.7% in lung cancer (27), and 78.6% in colorectal cancer (28). The relatively lower TLS rate in EMPD may be attributed to the fact that EMPD lesions are mostly confined to the epidermis, making it difficult to recruit the necessary cytokines to form TLS locally, particularly mature TLS (9.4%). This finding is consistent with studies of early melanoma, which similarly show challenges in forming TLS, as early melanomas are also predominantly confined to the epidermis with radial growth. For instance, primary melanomas demonstrated a TLS formation rate of 33.3%, with mature TLS present in only 8.3% (14), which aligns with our results. In another study of TLS in BCC, 63% of 30 samples from 16 patients exhibited TLS, though these were primarily immature or primary TLS, without any secondary TLS (20). This is comparable to our findings and suggests that tumors arising from the basal layer of the epidermis may similarly elicit weaker immune responses and less frequent formation of mature TLS. However, since BCC tumors are closer to the dermis, they may recruit cytokines more easily, accounting for the slightly higher TLS positivity rate compared to EMPD. In contrast, cSCC exhibited TLS in 87.8% of cases, with most being mature TLS (11). This may be explained by the fact that cSCC is more prone to dermal invasion and metastasis (29), leading to stronger immune stimulation and a higher rate of mature TLS formation. EMPD, on the other hand, remains primarily an epidermal tumor with limited invasion and metastasis, which could explain the lower presence of mature TLS. Due to the limited number of CD21-positive samples in our study, we did not perform additional staining for CD23 to further confirm the presence of secondary TLS (30).

In EMPD, TLS is primarily characterized by high density and peritumoral localization. Among TLS-positive patients, 88 cases (91%) showed high-density TLS. In contrast, cSCC exhibited high-density TLS in 78% of cases, where it was positively associated with better pathological differentiation. We speculate that the pagetoid spread of EMPD across the epidermis may lead to widespread but weak local immune stimulation, resulting in predominantly high-density, immature TLS. In contrast, cSCC typically infiltrates the dermis, leading to a more localized distribution but with greater TLS maturation. Due to the small number of EMPD cases with low-density TLS and the predominance of immature TLS, we did not investigate any potential correlation between TLS density and clinical outcomes. However, it is worth noting that higher TLS density has been significantly associated with poorer 10-year overall survival in primary colorectal cancer (31). Despite this exception, higher TLS density is generally correlated with improved survival across most tumor types.

Most studies on TLS localization report the presence of pTLS However, iTLS has been identified in various cancers, including hepatocellular carcinoma (HCC) (32), germ cell tumors (33), renal cell carcinoma lung metastases (34), intrahepatic cholangiocarcinoma (iCCA) (35), and cholangiocarcinoma (CAA) (36). In HCC, the presence of pTLS is associated with a higher risk of recurrence and poorer prognosis compared to iTLS. Similarly, in CAA, a high density of pTLS is linked to poor outcomes, while iTLS correlates with better prognosis (32). In iCCA, iTLS is negatively associated with tumor size and microvascular invasion, and is significantly linked to improved overall survival, whereas pTLS is positively associated with lymph node metastasis (35). These differences may arise from the distinct cellular compositions within and around the tumor, which could influence whether TLS exhibits pro-tumor or anti-tumor effects (11). In this study, only pTLS and sTLS were observed in epithelial EMPD, with no evidence of iTLS. This may be due to the intraepidermal nature of EMPD, which limits the recruitment of cytokines required to form TLS in the epidermis, while TLS can still develop in the dermis. Notably, we did not detect iTLS in invasive EMPD cases. No significant differences were observed between pTLS and sTLS regarding their impact on EMPD.

EMPD frequently invades skin appendages. Our findings show that patients with tumor cell infiltration of skin appendages are less likely to develop aTLS. This suggests that the presence of aTLS may predict good prognosis. In HCC (37) and colorectal cancer (38), where immature TLS is associated with higher tumor recurrence rates. This implies that TLS at early stages may hinder effective antitumor immunity, possibly due to the expression of immunosuppressive molecules. For TLS to support robust immune responses, a highly organized structure is required to facilitate optimal interactions between diverse immune cell types. This is underscored by findings in HCC, where intratumoral and peritumoral TLS containing mature GCs are associated with longer patient survival (39).

Nodule formation, tumor invasion, lymphovascular invasion, tumor thickness, and tumor location are recognized risk factors for EMPD (40). Our analysis showed no association between TLS presence and patient age, gender, common clinical manifestations, or subjective symptoms of EMPD. This finding is consistent with studies in BCC, where TLS prevalence was also unrelated to gender, age, or disease site (20). However, BCC with nodules were more likely to exhibit TLS, and in cSCC, sun-exposed areas correlated more strongly with TLS presence. Secondary EMPD was significantly more likely to develop TLS (84%) and mature TLS (69%), consistent with findings in melanoma (14). This may be because adenocarcinoma-derived tumors are typically not confined to the epidermis and tend to infiltrate deeper tissues, making them more likely to stimulate TLS formation. These metastatic tumors underscore the role of tumor origin in shaping the tumor immune microenvironment and influencing TLS formation from the outset. During metastasis, changes in the tumor microenvironment and established immunosuppressive mechanisms may impact TLS immune function. Recurrent EMPD was also more likely to develop TLS, particularly mature TLS. When examining changes in TLS between initial and recurrent cases in the same patients, we found that the presence of TLS increased from 53% to 82% following recurrence. This may reflect the body’s secondary immune response to tumor cells, leading to a stronger immune reaction and the formation of more abundant and mature TLS. In other tumor types, the density of immature TLS is generally not linked to prognosis, while mature TLS typically predicts a favorable outcome (19, 38, 41).

In our study, EMPD patients with tumor invasion were more likely to develop mature TLS, with most cases of mature TLS observed in tumors with a depth of infiltration of 4 mm or less. This suggests that tumor cell invasion into the dermis may promote the formation of mature TLS, which in turn may impede further tumor invasion, indicating a better prognosis. Vascular and lymphatic invasion did not appear to correlate with TLS formation in EMPD, possibly due to the limited sample size of mature TLS cases. Thus, future studies with larger sample sizes are necessary to validate these findings.

In this study, it was found that a large number of chemokines were highly expressed in EMPD, and a large number of T cells and B cells were infiltrated in EMPD tissues by inferential analysis of cell composition. TLS was found in EMPD, and mature TLS was associated with a good prognosis of invasive EMPD. In conclusion, the influence of a large number of chemokines in EMPD, infiltration of immune cells and mature TLS on the prognosis of invasive EMPD suggests the possibility of successful application of tumor immunotherapy in EMPD. Thus, inducing the formation of TLS in TME may be a promising strategy. However, there are still many doubts about the formation of TLS in tumors, the molecular mechanism of TLS-induced anti-tumor immune response remains to be further studied, and the mechanism of TLS formation in only some tumor tissues remains to be explored. It is worth noting that TLS, as a component of the immune system, can play an effective anti-tumor role and promote T cell transmission to inaccessible tumor areas, which may be a novel marker for good prognosis of tumor patients and contribute to the formulation of effective immunotherapy strategies and the development of new drugs (42, 43).

This study has several limitations. As limited RNA-sequencing data for EMPD can be available from the public database, only eight samples were used to assess the IME of EMPD, which might affect the accuracy. More samples should be sequenced for the IME evaluation in the future. There was also a lack of evaluation of how treatment affects TLS density/maturity, CD23 staining was not performed to identify secondary TLS, and the fact that our results are based only on evaluation of single slides of each antibody per case.

## 5 Conclusion

In EMPD, the TLS positivity rate was moderate, with the majority being high-density, immature, and located in the peritumoral area. Mature TLS, in particular, was more frequently observed in patients with secondary or recurrent EMPD. Patients with skin appendage infiltration were less likely to develop aTLS. *In situ* EMPD showed a lower likelihood of mature TLS formation, whereas invasive EMPD was more prone to developing mature TLS. Importantly, mature TLS was identified as a positive prognostic factor in invasive EMPD and may serve as a potential biomarker and therapeutic target.

## Data Availability

The datasets presented in this study can be found in online repositories. The names of the repository/repositories and accession number(s) can be found in the article/supplementary material.
