# Correction: The clinical and pathological significance of tertiary lymphoid structure in extramammary Paget’s disease

**DOI:** 10.3389/fimmu.2026.1784968

**Published:** 2026-01-21

**Authors:** Ningyuan Xi, Xiaoxiang Xu, Mingyuan Xu, Nanhui Wu, Yuhao Wu, Jiashe Chen, Shuyi Liu, Long Jiang, Guorong Yan, Guolong Zhang, Yeqiang Liu

**Affiliations:** 1 Department of Pathology, Shanghai Skin Disease Hospital, School of Medicine, Tongji University, Shanghai,, China; 2 Department of Phototherapy, Shanghai Skin Disease Hospital, School of Medicine, Tongji University, Shanghai,, China; 3 Department of Dermatologic Surgery, Shanghai Skin Disease Hospital, School of Medicine, Tongji University, Shanghai,, China

**Keywords:** extramammary Paget’s disease, tertiary lymphoid structures, histopathology, prognostic significance, skin cancer, non-melanoma skin cancer

There was a mistake in **Figure 1** as published. **Figure 1A** demonstrated the volcano plot regarding extramammary Paget’s disease (EMPD) vs. normal skin (NS) analyzed from GSE117285 data, which was an old version and the 10 significant chemokines were not highlighted. Therefore, we have highlighted the 10 significant chemokines in the new volcano plot of **Figure 1A** using the GSE117285 data. The corrected **Figure 1** appears below.

**Figure 1:**
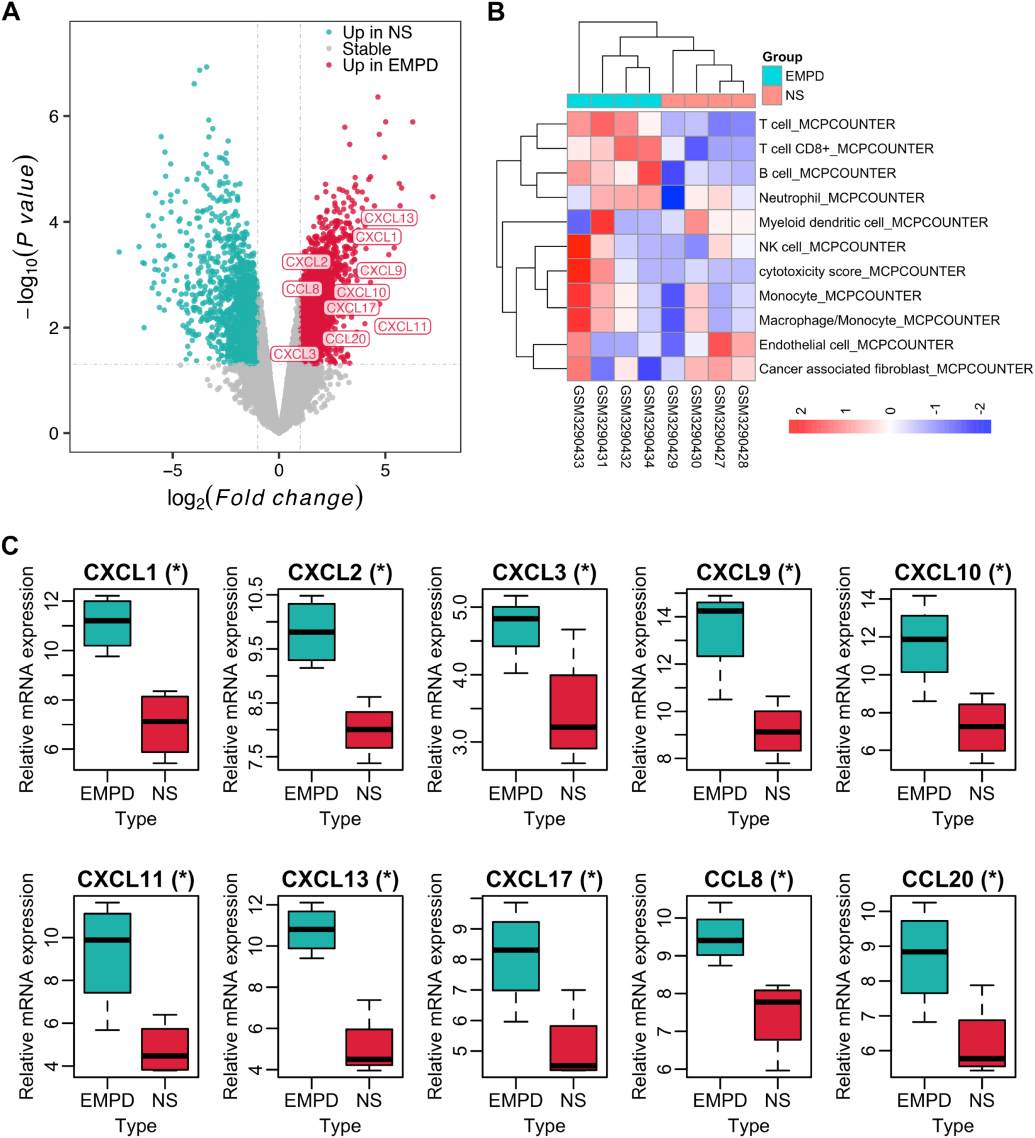
IME composition of EMPD and NS tissues in GSE117285. **(A)** Volcano plot for differentially expressed analysis in EMPD vs. NS tissue. **(B)** The IME composition in EMPD and NS tissues analyzed by the MCP-counter. NK cells, natural killer cells. **(C)** Differential expression of TLS-related chemokines in EMPD and NS tissue. NS, normal skin.

The original version of this article has been updated.

